# The Transcriptomics to Proteomics of Hair Cell Regeneration: Looking for a Hair Cell in a Haystack

**DOI:** 10.3390/microarrays2030186

**Published:** 2013-07-25

**Authors:** Michael E. Smith, Gopinath Rajadinakaran

**Affiliations:** 1 Bioinformatics and Information Science Center, Department of Biology, Western Kentucky University, Bowling Green, KY 42101, USA; 2 Department of Genetics and Developmental Biology, University of Connecticut Health Center, 263 Farmington Avenue, Farmington, CT 06030, USA; E-Mail: rajadinakaran@student.uchc.edu

**Keywords:** hair cell, regeneration, gene expression, microarray, transcriptomics, proteomics, microRNA, growth factors, inner ear

## Abstract

Mature mammals exhibit very limited capacity for regeneration of auditory hair cells, while all non-mammalian vertebrates examined can regenerate them. In an effort to find therapeutic targets for deafness and balance disorders, scientists have examined gene expression patterns in auditory tissues under different developmental and experimental conditions. Microarray technology has allowed the large-scale study of gene expression profiles (transcriptomics) at whole-genome levels, but since mRNA expression does not necessarily correlate with protein expression, other methods, such as microRNA analysis and proteomics, are needed to better understand the process of hair cell regeneration. These technologies and some of the results of them are discussed in this review. Although there is a considerable amount of variability found between studies owing to different species, tissues and treatments, there is some concordance between cellular pathways important for hair cell regeneration. Since gene expression and proteomics data is now commonly submitted to centralized online databases, meta-analyses of these data may provide a better picture of pathways that are common to the process of hair cell regeneration and lead to potential therapeutics. Indeed, some of the proteins found to be regulated in the inner ear of animal models (e.g., IGF-1) have now gone through human clinical trials.

## 1. Introduction

Hearing and vestibular impairment can be caused by the loss of sensory hair cells in the inner ear of vertebrates. These hair cells can be damaged and/or lost by exposure to loud or prolonged sound [1,2,3], ototoxic drugs [4] and disease and aging [5]. Hair cell loss in humans and other mammals is permanent, but regeneration of lost hair cells occurs spontaneously in non-mammalian vertebrates, including fish [6,7,8,9], amphibians [10,11], reptiles [12] and birds [13,14,15,16] (also, see review in [17]). This potential for the production of new hair cells in non-mammalian vertebrates has been known since the 1930s, when the regeneration of the lateral line hair cells on amphibian tails was studied [18,19]. Later, in the 1980s, it was discovered that new hair cells were formed in the inner ears of adult cartilaginous and bony fishes [20,21,22] and that birds could regenerate new hair cells following damage-induced loss of hair cells in the basilar papillae [13,14,15,16,23].

Since these earlier discoveries, researchers have been examining the process of hair cell death and regeneration in non-mammalian animals, in an attempt to find ways of stimulating the production of new hair cells in mammals. The ultimate goal of this research is to find new therapeutics for human sensorineural hearing and balance deficits. Recent advances in molecular biology techniques have assisted in this search. Gene expression profiling of inner ear tissues has been useful in discovering cellular pathways that are regulated during the process of hair cell death and regeneration. With this technique, scientists have identified genes that are up- or downregulated in comparison to different time points following trauma to the inner ear [24,25,26] or between sensory tissue types that differ in terms of normal cell proliferation (e.g., the mammalian utricle* vs.* cochlea) [27] or between different cell types within a single sensory organ [28]. 

The technologies for examining such patterns have evolved rapidly—from dot blots to microarray analysis, to next-generation sequencing (NGS) and microRNA analysis [29]. Simultaneously, techniques for protein separation and characterization have also improved, increasing the role of proteomics in understanding processes in the inner ear [30]. This article reviews the methods that have been used in an attempt to find cellular pathways that are regulated during the process of hair cell regeneration in vertebrates and discusses the results of representative studies using these methods in an effort to find common pathways important to hair cell regeneration and potential therapeutic targets to treat deafness and balance disorders.

## 2. Experimental Methodologies

**Figure 1:**
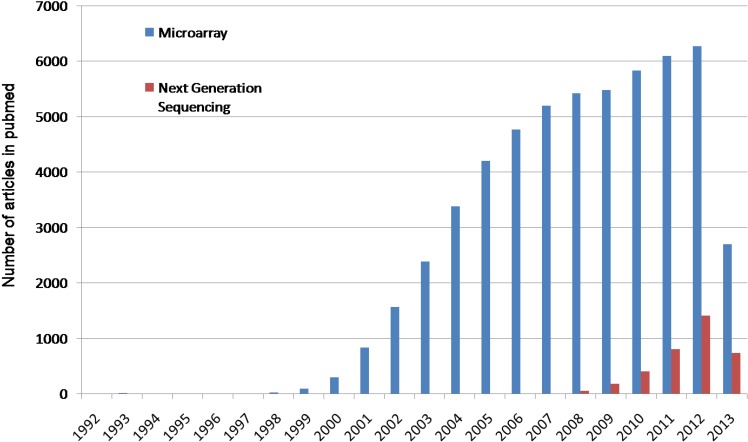
Graph indicating the number of articles in PubMed including one of the two keywords “microarray” or “next-generation-sequencing” in their articles.

The state of a cell can be understood by the genes that are transcribed at a given time. Gene expression profiling studies (*i.e.*, transcriptomics) are tremendously useful in providing a snapshot of what mRNA transcripts are made in the cell. Both microarray and NGS technology are widely used to understand the expression of genes under varying conditions (disease, developmental time points, following stress,* etc.*). A PubMed search with either the key word “microarray” or “next-generation-sequencing” showed that the reference of these technologies has increased tremendously in the last decade, from only a few papers in the late 1990s to over 7,000 publications in 2012. NGS technology is a relatively newer subset of these publications and has increased considerably since 2008 (Figure 1). Each microarray or NGS study can result in huge datasets of hundreds to thousands of differentially expressed transcripts, which has led to an explosion of new data. As a result, looking for cellular pathways specific to hair cell regeneration can be like looking for a hair cell in a haystack, so to speak. Fortunately, bioinformatics tools are being developed alongside molecular tools to help filter all the rapidly growing data and assist in finding the most important cellular networks of interest.

Microarray technology evolved from the traditional dot blot technique that was used for parallel screening of small numbers of genes, and it works by the same principle of hybridization between the target DNA and the probe [31,32]. Complementary DNA (cDNA) and oligonucleotide arrays are two miniaturized forms of dot blot that can be used to identify thousands of genes at the same time. In the cDNA array, a control and an experimental sample are fluorescently labeled separately using different dyes and applied on the same array to determine the relative intensities of genes printed on the chip. Later, with the development of oligonucleotide arrays, the need for adding a control sample along with the experimental sample was overcome [32]. This array technology has been widely used in many different organisms, tissues and* in vitro* culture studies relevant to the biology of the inner ear (Table 1). TaqMan low density arrays are another type of array that are based on the real-time quantitative reverse transcription-PCR (QRT-PCR) method to study more focused gene expression patterns in tissues. This method is more sensitive than gene chips and offers higher throughput than standard QRT-PCR [33]. Serial analysis of gene expression (SAGE), another technique used to identify genes that are differentially expressed, is based on unique tag sequences that are sufficient to identify different transcripts [34]. Later, the advent of NGS technology completely changed the way of analyzing gene expression data. Unlike microarray, this technology works by identifying genes by sequencing fragmented short reads. Both SAGE and NGS eliminate the need for prior knowledge of probes to scan for genes and, thus, have the potential to identify alternative splice variants and novel transcripts, with greater sensitivity for the transcripts that are expressed at low levels. NGS comes in different platforms that vary in sequencing chemistry and can be applied to study questions at different biological levels relating to the transcriptome or epigenome [35,36]. While the transcriptome is the set of transcribed RNAs that are found in a tissue, the epigenome examines regulation of gene expression via DNA cytosine methylation or deamination, histone protein modifications, such as acetylation, genomic imprinting and RNA editing. Understanding of the role of epigenetics in hair cell development or regeneration is still in its infancy, but early studies have been reviewed elsewhere [37].

**Table 1:** Representative studies examining gene expression in the inner ear during development, in different cell/tissue types and following trauma.

Reference	Organism and organ	Methodology	Developmental stage or treatment
*Developmental differences in gene expression*			
[38]	Mouse cochlea	Affymetrix GeneChip oligonucleotide array	P2 and P32
[39]	Mouse cochlea	Affymetrix GeneChip oligonucleotide array	P2 and P32
[40]	Mouse cochlea-conditionally immortal cell line	Affymetrix GeneChip oligonucleotide array	14 days following differentiation
[41]	Mouse inner ear	Affymetrix GeneChip oligonucleotide array	E9-E15
[42]	Mouse cochlea	Affymetrix GeneChip oligonucleotide array	P3 and adult
*Cell/Tissue differences in gene expression*			
[27]	Chick cochlea and utricles	Custom built cDNA and oligonucleotide arrays	cochlea * versus* utricles
[28]	Rat cristae ampullaris	Agilent RNA6000 Nano Lab Chip	hair cells * versus* supporting cells
[41]	Mouse inner ear	Affymetrix GeneChip oligonucleotide array	cochlea, utricle, or saccule
[24]	Chick inner ear	Custom built TF oligonucleotide array	30 min and 1, 2, 3 h post-ototoxic or laser trauma
[43]	Zebrafish lagena	Affymetrix GeneChip oligonucleotide array	hair cells * versus* liver hepatocytes
*Gene expression during regeneration following trauma*			
[24]	Chick inner ear	Custom built TF oligonucleotide array	30 min and 1, 2, 3 h post-ototoxic or laser trauma
[25]	Zebrafish inner ear	Agilent Zebrafish oligonucleotide arrays	2 and 4 days post-acoustic trauma
[44]	Zebrafish inner ear	Illumina tag profiling (SAGE)	0, 1, 2, 4 days post-acoustic trauma

Both microarray and NGS have been applied to understand processes related to hair cell regeneration in the inner ear of vertebrates. Three basic experimental paradigms have been utilized. (1) Since the process of regeneration and development may have redundant pathways, understanding temporal shifts in gene expression patterns during the normal development of the auditory system and the production of new hair cells may provide clues to important cellular pathways used for hair cell regeneration. (2) Another clue to processes involved in hair cell regeneration is gene expression patterns between auditory sensory tissues that differ in their ability to regenerate. The mature mammalian sensory epithelium of the cochlea do not form new hair cells post-development or following hair cell damage or loss, but maintain mitotic quiescence [45,46]. In contrast, the mammalian vestibular epithelium exhibits more plasticity, and damage to the utricle has led to supporting cell proliferation [47] and even some cells differentiating into cells with hair cell-like characteristics [48,49]. Thus, gene profiling studies contrasting differences between quiescent and regenerating tissues may bring to light pathways that are activated during hair cell regeneration. (3) Lastly, the most direct method of examining genes and pathways important during hair cell regeneration is to damage the inner ear with either ototoxic chemicals, acoustical overstimulation or mechanical ablation and, then, analyze shifts in gene expression at specified time points during the recovery process. Some representative studies that have used these three experimental paradigms during the last decade are summarized in Table 1. While much of the focus has been on gene expression patterns (transcriptomics) as a means of understanding important cellular processes involved in hair cell regeneration, regulation of these processes can also occur at the post-transcriptional, translational and post-translational levels. In fact, mRNA levels often do not correspond well with protein expression levels, because protein abundance is dynamic [50]. For example, some proteins are constitutively expressed, and their functionality is mediated by phosphorylation or other post-translational events. In addition, mRNA levels cannot predict post-translational modifications or which splice variants are translated. 

As a result, there has been a surge in research on post-transcriptional control (primarily examining microRNAs) and proteomics in the inner ear. The study of proteomics started from 2D gel electrophoresis in which the proteins are first separated in the first dimension by their isoelectric point and, then, in the second dimension by their mass. The separated proteins can then be cut out, in-gel trypsin digested and analyzed using mass spectrometry. Using 2D-difference gel electrophoresis (2D-DIGE), different experimental samples can be labeled separately and mixed together before isoelectric point separation, similar to sample preparation for microarrays [31,51]. Large-scale protein analysis can be studied by using protein arrays that contain antibodies attached to the chip to detect its specific antigens [52]. Liquid chromatography in tandem with mass spectrometry (LC-MS) can be used to identify large numbers of proteins, and it can detect low molecular proteins that are less than 5,000 KDa [31]. The post-translational modification of proteins can be identified from such proteomic techniques, but not from gene microarrays. 2D-DIGE and LC-MS methods have been used to study the inner ear proteome [53,54,55,56,57]. Together, large-scale transcriptome, microRNA and proteome studies have provided and continue to provide copious amounts of data to mine in order to understand the process of hair cell regeneration in the inner ear at both the transcript and protein level. 

## 3. Gene Profiling in the Vertebrate Inner Ear

### 3.1. Developmental Shifts in Gene Expression

Since it has been hypothesized that regeneration recapitulates development, understanding temporal shifts in gene expression patterns during the normal development of the auditory system may provide clues to important cellular pathways used for hair cell regeneration. One of the first two studies to use microarray technology to examine gene expression in the inner ear was by Chen and Corey [38,39]. They examined mouse cochleae at two developmental stages (postnatal days 2 and 32) to find differential gene regulation between developing and mature (quiescent) auditory tissues. Since then, gene expression at a number of other developmental stages has been studied in the mouse ear (e.g., E9–E15 [41], P3 and adult [42]). Similarly, Rivolta* et al.* [40] quantified the time course of gene expression following induced differentiation in conditionally-immortal cells derived from mouse cochleae. These microarray studies confirmed previous evidence that key signaling pathways, such as *Notch* and *Wnt*, are important to inner ear development. 

For example, *Notch1 *and* Notch3*, as well as downstream effectors of the notch cascade, such as *Hes1* and *Hes3*, were significantly regulated during differentiation of cochlear cells [40]. *Nrarp* (Notch-regulated ankyrin repeat protein), which is thought to be part of a negative feedback pathway to attenuate Notch signaling, was upregulated in P3 mouse cochlea relative to adult tissue [42]. A number of *Wnt* genes were expressed in P2 and/or P32 mouse cochleae, including *Wnt-4*,* Wnt-5a*,* Wnt-5b*,* Wnt-7b* and *Wnt10a* [38], but the roles of different* Wnt* genes vary during development. Four *Wnt* genes were upregulated only in the early developmental stages of the mouse ear, while eleven were upregulated only in the later stages of inner ear development [39].

Microarray studies have also confirmed the importance of cell cycle regulation genes, such as those for cyclin-dependent kinase inhibitors for inner ear development. For instance, *p27Kip1*, *p27Kip2*, *p19Ink4d* and *p15Ink4b* [38,40,41] were regulated during development of the mouse inner ear. In general, these genes were downregulated in early developmental stages during significant cell proliferation and upregulated in later stages during cell differentiation [40,41]. 

The power of microarray analysis goes beyond verifying genes that are already known to be expressed in the inner ear, to establishing networks of genes and finding novel genes and pathways. Some examples of such novel genes and pathways discovered via gene expression analysis to be regulated in the inner ear during development include semaphorins [40], *Hmga2* (high mobility group AT-hook 2), *Nrarp*, *Prl* (prolactin) and *Ar* (androgen receptor) [42] and circadian rhythm and estrogen receptor signaling pathways [41].

### 3.2. Cell- and Tissue-Specific Transcript Profiling

One of the first steps to understanding pathways involved in hair cell regeneration is to separate genes that are expressed in auditory sensory tissues compared to other reference tissues. One of the first array studies to be applied to inner ear tissue compared regions of the rat cochlea to the cochlear nucleus, inferior colliculus and hippocampus [58]. Greater differences in gene expression were found between the cochlea and the central nervous system regions than between the central auditory regions and the hippocampus, showing that gene expression patterns in peripheral and central nervous tissues differ. Genes that were expressed at higher levels in the cochlea included insulin-like growth factor binding proteins, matrix metalloproteinases and tissue inhibitors of metalloproteinases [58].

Within the inner ear itself, gene expression can vary among different end organs (*i.e.*, cochlea, utricle, saccule and cristae in mammals). Sajan* et al.* [41] found unique gene expression signatures for the mouse cochlea, utricle and saccule. This is relevant to hair cell regeneration research, because there is evidence for limited hair cell regeneration in the mammalian utricle [47,48,49], but not in the cochlea. Thus, differences in gene expression between the separate auditory end organs are not surprising. In contrast to the mammalian utricle, the avian utricle is in a constant process of apoptosis and regeneration [59,60,61,62]. The avian cochlea is similar to the mammalian cochlea, though, in that it is normally in a quiescent state [63]. Thus, comparing gene expression between different end organs may highlight potential therapeutic targets that may help guide mammalian cochlear sensory epithelia into a proliferative state, allowing for potential hair cell regeneration. Hawkins* et al.* [27] found 20 different inner ear genes and 80 transcription factors (TF) that were significantly different between the avian cochlea and utricle. *Bmp4*, *Gata3*, *Gsn*, *Foxf1* and *Prdm7* were some of the genes that were upregulated in the cochlea, while *Smad2*, *Kit*, *β-amyloid*, *Loc51637*, *Hmg20b* and *Crip2 *are examples of genes that were upregulated in the utricle. While some of these genes are well known to be involved in the development of the inner ear (e.g., Gata3 [64]), some of them were novel TF, like *Loc51637* and *Hmg20b*, about which little was previously known.

At an even finer scale, the transcriptome of hair cells can be examined. Cristobal* et al.* [28] used laser capture microdissection to collect hair cells and supporting cells separately from the rat cristae to compare expression profiles between the two cell types. There were 97 and 78 annotated genes with greater than a five-fold expression difference in hair cells relative to supporting cells and supporting cells relative to hair cells, respectively [28]. Another means of separating hair cells from supporting cells for a pure hair cell transcriptome is to dissociate them from the sensory epithelia using proteases. McDermott* et al.* [43] isolated a population of pure hair cells from the zebrafish lagena and compared the hair cell transcriptome to that of control liver tissue. They found 1,037 hair cell-specific genes supporting a range of functions, including synaptic transmission, transcriptional control, membrane transport, cellular adhesion, cytoskeletal organization and signal transduction, as well as candidate deafness genes, such as *KIDINS220*. 

### 3.3. Gene Expression Following Inner Ear Trauma

A number of microarray studies have examined gene expression following trauma to the non-regenerative mammalian cochlea. These and other inner ear microarray studies are more thoroughly reviewed by Hertzano and Elkon [29]. Gene expression in mammalian (non-regenerative) models can be compared to shifts in gene expression patterns following trauma to the non-mammalian (regenerative) inner ear to highlight functional pathways involved in hair cell death and regeneration. Although the process of regeneration of adult inner ear tissue may recapitulate some of the same processes of initial sensory epithelial development, it is highly likely that there are important differences, as well. Thus, measuring gene expression in tissues that are going through the regeneration process is the most direct way to discover what pathways are activated during hair cell regeneration. 

Trauma to the ear can be produced by ototoxic chemicals [24], acoustic overstimulation [25,44,65] or laser ablation [24]. Hawkins* et al.* [24] performed the first large-scale microarray experiment on regenerating auditory tissues. They examined gene expression of TF in cultured avian utricles and cochleae following trauma induced by either a pulsed laser microbeam or the ototoxic antibiotic, neomycin. Although there were differences in expression patterns between tissue types and treatments, there were a number of identical gene expression patterns found across treatments during the process of regeneration. Some of the identified signaling pathways were *TGFβ*, *PAX*, *NOTCH*, *WNT*, *NFKappaB*, *INSULIN/IGF1* and *AP1*. In addition, *p27^KIP^* and genes that regulate its expression and other apoptotic and cell cycle control pathways, were significantly regulated during regenerative proliferation.

Following noise exposure, Schuck* et al.* [25] examined microarray gene expression patterns in zebrafish ears at two and four days post-exposure. Transcripts that showed the greatest regulation on day 2 compared to control tissues included growth hormone 1 (*gh1*, upregulated) and major histocompatibility complex, class I, ZE (*mhc1ze*, downregulated). Many genes that were upregulated on day 2 were downregulated at day 4 and *vice versa*. Follow-up experiments showed that growth hormone (GH) injection following acoustic exposure led to an increase in cell proliferation and a decrease in apoptosis in the zebrafish inner ear [65]. Insulin-like growth factor 1 (IGF-1) is secreted mainly by the liver and is stimulated by GH. It is required for normal post-natal survival, maturation and differentiation of cochlear ganglion and hair cells [66]. Thus, it is likely that the effects of GH on the zebrafish inner ear are also mediated by IGF-1. A number of genes involved with immune function, including MHC class I and II molecules, were also significantly regulated in the zebrafish ear post-acoustic exposure. These genes may play a role in cell proliferation following hair cell death. When MHC Class I molecules are bound by antibodies, it prevents them from presenting antigens and promotes cell proliferation [67]. Thus, the downregulation of *mhc1ze* may have promoted increased cell division, as it coincides with a peak in cell proliferation in the zebrafish ear following acoustic trauma [9].

Liang* et al.* [44] also examined gene expression in the zebrafish ear after noise exposure, but at more time points (immediately after two days of exposure and one, two and four days post-exposure). They used digital gene expression (DGE), which utilizes tag sequence profiling. Immediately after noise exposure, *stat3 *and* socs3a* were significantly upregulated and the *stat3*/*socs3a* pathway was the dominant signaling pathway that was regulated. Genes related to this pathway were also significantly regulated (e.g., *socs3b*, *jak1*, *mmp9*). Interestingly, the same or similar genes were found regulated in the zebrafish ear by Schuck* et al.* [25]. These included *socs3b*, *socs1, mmp13* and *stat1b*. The *stat*/*socs* pathways are activated by GH [68], so it makes sense that these genes are regulated as GH is upregulated following acoustic trauma to the ear. 

## 4. Role of MicroRNAs in the Development, Maturation and Functioning of Hair Cells

### 4.1. Introduction to the Role of MicroRNAs in the Inner Ear

MicroRNAs (miRNAs) are small (~22 nucleotides-long), non-coding RNAs that regulate gene expression either by translation repression or mRNA destabilization or both [69]. miRNAs can be transcribed from either the introns of genes or from independent transcription units. In mammals, the primary transcript of miRNA, called pri-miRNA, is first cleaved by Drosha RNAse III endonuclease giving rise to precursor miRNA (pre-miRNA), a ~60–70 nucleotides-long stem-loop structure. This pre-miRNA is then transported to the cytoplasm, where it is again cleaved by another enzyme Dicer, leaving an imperfect double-stranded RNA. This double-stranded RNA is then loaded into the RNA Induced Silencing Complex and cleaved into a single-stranded mature miRNA containing the seed region (2–7 nucleotides) to target the mRNA transcripts that have either perfect or imperfect complementary sequences in their 3′ UTR regions [70]. The seed regions of miRNAs are highly conserved between many organisms [71]. miRNAs can regulate many cellular functions, such as cell proliferation and apoptosis, owing to their binding potential to many target mRNAs [70,72]. Dicer knockout studies in mice [73] and zebrafish [74] showed reduced mature miRNAs, indicating that Dicer is crucial in processing pre-miRNAs to mature miRNAs. miRNAs can occur as clusters in the genome, and over half of the known miRNAs in the *Drosophila* genome are clustered, although this is not the case with the human and worm genome, where only a small fraction of miRNAs are clustered,* i.e.*, many are found to be isolated [70,75]. The regulation of miRNAs is dynamic, and they show remarkable variability in abundances in cells. For example, in the adult worm, miR-2 is present at more than 50,000 copies, whereas miR-124 is present at ~800 copies per cell [76]. 

A large number of miRNAs have been shown to affect hair cell development in zebrafish [77]. Interest in understanding the role of miRNAs in regulating mechanosensory hair cells has increased ever since the first identification of expression of miRNAs in the sensory epithelia of zebrafish [74]. Following this, many groups have started to explore the developmental and functional role of miRNAs in the inner ear of model organisms, including zebrafish [73,76], mouse [71,73,78,79,80,81,82], rat [83], chicken [84,85] and salamanders [86]. 

Microarrays can be used to detect a large number of miRNA molecules at a time. During the early development of the inner ear, both at the embryonic and post-natal stages, a large number of miRNAs have been detected [74,78,79,86]. Within the inner ear, miRNAs show regulation at both spatial and temporal levels [70,73,74]. For example, in mouse, miR-99a is expressed in hair and supporting cells in the cochlea, but it is expressed only in hair cells in the vestibule [73]. In zebrafish, the majority of the miRNAs examined at the embryonic stage showed very little expression, but they were expressed after the completion of organogenesis, suggesting that miRNAs are required during development, but not during embryonic growth [74]. Differential expression of miRNAs within different inner ear structures have been documented [73,78]. Microarrays done in newborn mouse inner ear showed that 24 miRNAs were differentially expressed between cochlear and vestibular structures [73]. Another study found differential expression of over 100 miRNAs in five developmental time points [78]. Table 2 lists differentially expressed miRNAs that have been found in the inner ear of model organisms. In order to understand the importance of mature miRNAs in the inner ear of mammals, some studies have knocked out the enzyme, Dicer, which processes pre-miRNA to mature miRNA. A conditional Dicer*^fl^°^x/fl^°^x^* knockout mouse was generated by expressing cre recombinase under the *Pou4f3* promoter to study its role in hair and supporting cells of the mouse inner ear [73]. 

**Table 2:** List of differentially expressed microRNAs found in different organisms and tissues detected using microarray analysis.

Micro RNA	Organism	Reference	Organ of expression
miR182, miR183, miR96	Zebrafish	[74]	Hair cells of neuromasts and inner ear, nose, cranial ganglia, eye, epiphysis
miR183, miR182, miR96	Mouse	[78]	Inner ear
miR141, miR200a, miR200b, miR139	Zebrafish	[74]	Neuromast, nose, epidermis, taste buds, proctodeum
let7g, miR15a, miR17-5p, miR18, miR19a, miR19b, miR20, miR210, miR25, miR26a, miR26b, miR92, miR93	Zebrafish	[74]	Neuromasts, head, spinal cord, gut, somites
miR15a1, miR18a	Zebrafish	[73]	Neuromasts, hair cells, otocyst and head
miR199a	Mouse	[73]	Cochlea
miR99a, miR15a, miR30b	Mouse	[73]	Cochlear hair and supporting cells, vestibular hair cells, spiral ganglion neurons and basilar membrane
miR18a	Mouse	[73]	Cochlea, Spiral ganglion neurons, vestibular hair and supporting cells
Let7a, let7b, let7c, let7d, let7e, let7f, let7g	Mouse	[73]	Cochlea and vestibule
Let7i	Mouse	[73]	Vestibule
Let7a, let7b, let7c, let7d, let7e, let7f, let7g, let7i	Newt	[86]	Inner ear
miR9, miR124a	Mouse	[78]	Inner ear, brain
miR10a, miR107, miR124, miR130b, miR146b, miR183, miR190b, miR200c, miR30d, miR30e, miR325, miR333, miR339-3p, miR381, miR429, miR532-3p, miR674, miR99b, miR194, miR186 and miR331-5p	Rat	[83]	Cochlear epithelia
miR182, miR140	Mouse	[79]	Otocyst, spiral ganglion, inner and outer hair cells, utricle, saccule, crista
miR194	Mouse	[79]	Hair cells of cochlea and vestibule, spiral ganglia
miR376a, miR376b	Mouse	[80]	Otic placode, organ of Corti, spiral ganglia, stria vascularis, ampulla of vestibular organs
miR135	Mouse	[87]	Hair cells in vestibule, vestibular neurons and spiral ganglia
miR205	Mouse	[87]	Cochlea, spiral ligament, basilar membrane, apical surface of the spiral limbus

No phenotype was observed in the inner ear in the early post-natal day 0 (P0) stage, but at P38, after maturation of hair cells in the cochlea, severe malformations were observed in the hair cells in the base, while a less severe phenotype was observed in the apex. Scanning electron microscopy in the cochlea and vestibule also revealed the ultrastructural changes, including loss of stereocilia, hair cells becoming round in shape and disorganized and fused stereocilia [73]. Another study knocked out Dicer conditionally in mouse using the *Pax2* promoter, and profound morphological and developmental defects were observed in the early embryonic stages [81]. The phenotypes included smaller otocysts, absence of horizontal and anterior cristae, reduced saccules and utricles, reduced inner ear innervation and disorganized hair cell stereocilia. A third study using *Atoh1-cre* mouse for knocking out Dicer found a marked reduction in the number of outer hair cells and, to some extent, in the inner hair cells [88]. These studies utilized different promoters for knocking out Dicer, but all found severe defects in the development of the inner ear, suggesting that mature miRNAs play an essential role in the morphogenesis, development and innervation of hair cells.

### 4.2. Important miRNA Clusters in the Inner Ear

One of the most widely studied miRNA groups is the miR-183/182/96 cluster [73,74,77,78]. miR-183, miR-182 and miR-96 are expressed from the cluster as a polycistronic unit in the mouse inner ear [78]. miR-183 controls the differentiation of hair cells [77], while mutations in the miR-96 seed region are associated with hearing disorders [89]. This cluster showed strong expression of mature miR-183, miR-182 and miR-96 in the hair cells of both mouse cochlea and vestibule at P0, but the levels of pri-miR-183/96 [78] and miR-183 [88] varied between the hair cells in the apex and base of the cochlea. This signifies that hair cells at different locations in the inner ear may process miRNAs differently. Expression of the miRNAs from this cluster has also been identified in the embryonic stages of the mouse inner ear [79] and in zebrafish hair cells and neuromasts [74], indicating a high degree of conservation between organisms. Interestingly, the expression pattern of miR-182 was broad in many embryonic tissues, but confined specifically to the inner ear during post-natal periods [79]. In mouse, the levels of miR-183, miR-182 and miR-96 changed over developmental time between P0 and P100. Notably, miR-183 and miR-96 were upregulated during adult stages, while miR-182 was not [78]. Another group identified the expression of antisense miR-182 in the mouse cochlea and vestibule at P0, suggesting potential downregulation of miR-182 in the inner ear [73]. Inhibition of miR-183 in zebrafish using morpholinos decreased the number of hair cells in sensory macula in both the inner ear and neuromasts [77]. Overexpression of either miR-182 or miR-96 in zebrafish embryos showed duplicated otic vesicles and an increase in the number of ectopic hair cells, suggesting that these miRNAs can control hair cell fate during development. Morpholinos targeted towards either miR-182/miR-183, miR-96 or miR-183/182/96 all decreased hair cell numbers in the zebrafish inner ear [77].

Let-7 family members are another class of widely studied miRNAs in the inner ear, and members of this family specify transition of cells from larval to the adult stage in *C. elegans* [90]. In salamanders, let-7 family members were found to be regulated during regeneration of a labyrinth culture* in vitro* [86]. Treatment of adult newt labyrinth with gentamicin caused hair cell death, and gene expression studies indicated let-7g and let-7c were downregulated while let-7a and let-7e were upregulated at day 7 following gentamicin treatment. All members from let-7a through let-7g were found to be downregulated at day 12 [86]. Adult mice exposed to acoustic overstimulation showed expression of let-7a, 7b, 7d, 7e, 7f, 7g and 7i [83]. Since both antibiotic treatment and acoustic overexposure regulate let7 miRNAs, this indicates a common pathway for hair cell degeneration. miRNA profiling in normal aging mice revealed upregulation of let-7a, 7b, 7c, 7e, 7f, 7g and 7i miRNAs, suggesting they may regulate pro-apoptotic pathways, leading to degeneration of hair cells [82]. In another study, let-7a and let-7b showed opposite expression profiles between cochlea and vestibule in the newborn mouse [73]. During the development of the mouse inner ear, members of let7 family (let7a–let7i) were differentially expressed between P0 and P100 [78]. An interesting observation from this study is that all the let7 members were downregulated at the P21 developmental time point, while most let7 members were upregulated in the P35 and P100 adult stages. Let-7e was downregulated at all five time points analyzed, suggesting it may be a negative regulator for the growth of auditory sensory epithelia [78].

MiR-181 is known to cause proliferation of hair cells in the chicken inner ear and inhibition of miR-181a reduces proliferation [85]. Consistent with this, overexpression of miR-181a in the chick basilar papillae* in vitro* showed an increase in the number of hair cells, and this proliferative effect can be enhanced by the addition of forskolin [84]. Upregulation of miR-181a and miR-181b in the chick inner ear was found at P0 and P8, and it gradually decreases towards the adult stage [78]. miR-181a, miR-181c and miR-181d were downregulated in two normal aging mouse models, C57BL/6J and CBA/J [82], implying that aging can lead to decreased levels of miR-181, which then inhibits proliferation. This suggests that during the early post-natal growth of the inner ear, miR-181 is required, but may not be necessary during the adult stage. However, adult rats showed expression levels of miR-181a that were different from the mouse inner ear, suggesting species-specific differences may also exist [83]. 

Acoustic trauma caused downregulation of miR-183 approximately three-fold in rat inner ear [83]. Age-related hearing loss was associated with the downregulation of miR-183 at three months of age in C57BL/6J mice and at nine months in CBA/J mice [82]. The discrepancies in the onset of downregulation of miR-183 can partly be attributed to the different genetic backgrounds of these mouse models. C57BL/6J mice have the presence of the *Ahl *gene, which causes deficiencies in cadherin23. This suggests that interference with genes essential for normal hair cell functioning can cause rapid loss of hair cells in these models. miR-183 was downregulated in the cochlea of acoustically-overexposed rats, and then, computational analyses predicted that this downregulation could be potentially associated with cell death and apoptotic functions [83]. 

Mutations in the seed regions of miRNAs are reported to segregate with hearing disorders and cause Mendelian diseases [71,89]. Any changes to the conserved seed region in the miRNA cause disruptions in base pairing to its target mRNAs. A study of Spanish patients reported mutations identified in the seed region of miR-96 in the fourth (G > A) and fifth (C > A) nucleotides that are highly conserved between 14 different organisms. These mutations were associated with hearing disorders [89]. The diminuendo (*Dmdo*) mouse model has an *N*-ethyl-*N*-nitrosourea-induced mutation at the sixth nucleotide (A > T) within the seed region of miR-96. This mutation caused a severe hair cell degeneration phenotype in both heterozygotes and homozygotes, with the latter having a quicker onset [71]. Mutations in miR-96 caused a shift in the global gene expression profile with almost 90 direct, indirect and acquired target genes. A novel mutation in the stem region of miR-96 (+57T > C) was identified in non-syndromic sensorineural hearing loss patients [91]. Although this mutation is not in the seed region, it was reported to decrease the levels of both miR-96 and antisense miR-96. 

In summary, miRNAs can regulate mRNAs at the post-transcriptional level by binding to their target molecules. Global change in miRNA expression profiles have been documented with both pre- and mature forms during development [78], acoustic exposure [83] and antibiotic treatment [86]. Studies using conditional Dicer knockout in the mouse inner ear clearly demonstrated that mature miRNAs are important for the morphology and development of hair cells. A single miRNA can have multiple target mRNAs, which indirectly controls the expression of many genes and affects many cellular pathways. Bioinformatic analyses have revealed many potential targets for miRNAs expressed in the inner ear that can be categorized into functions, such as cell death, cell proliferation, RNA metabolic processes, Wnt signaling and protein kinase cascades [83]. For example, the targets for miR-96 were identified to be *Aqp5*,* Celsr2*,* Odf2*,* Myrip *and* Ryk* [71] and for miR-182, *Sox2* [88], *Egr1*, *Irs1* and *Taok1 *[83], using luciferase assays. Reduced Sox2 levels in the mouse inner ear causes hearing impairments and severe malformations [92]. It is quite clear from these studies that non-coding miRNAs play an important role in regulating mRNA transcripts post-transcriptionally. Currently, only a handful of targets are known for miRNAs. Although profiling studies and computational analyses can predict mRNA targets, further experimental validation is required to show if they are indeed true targets of the miRNAs of interest. 

## 5. Proteomic Analysis of the Inner Ear

miRNAs can affect their targets either by degrading the mRNA or by repressing their translation [70]. If miRNAs target their mRNAs at the translational level by causing repression, it is not possible to identify those targets by profiling their gene expression, as the mRNA levels will remain the same, while the protein levels change. The reason why proteomics is necessary can be easily understood by comparing the ~30,000 human coding genes and the ~500,000 proteins resulting from post-translational modifications (PTMs), splicing variants and proteolysis products resulting from these genes [30,50]. Furthermore, the relative differences between mRNA levels and protein turnover, differences in the function of translational machinery and complex interactions cannot be understood from gene array studies [50]. Proteomic profiling done in the inner ears of mouse have identified that the targets of miR-135 and miR-205 only change at the protein level, but not at the mRNA level [87]. A comparison of mRNA and protein profiling did not show complete correlation between transcript and protein levels, indicating that protein expression levels cannot always be extrapolated from mRNA levels [57,93]. 

### 5.1. Antibody Microarrays

Proteins can be separated and characterized via a number of different techniques (reviewed in [30]). One of these techniques is the use of antibody microarrays, which are similar to DNA microarrays, except that antibodies are printed on the chip instead of DNA probes [52]. Antibody microarrays were first used by Jamesdaniel* et al.* [53] to study hair cell damage in the inner ears of rats treated with cisplatin. This study identified the dynamic changes of 19 proteins that were either up- or downregulated and 15 of those were novel proteins that belong to either cell death or survival pathways [53]. Differential expression of proteins in the organ of Corti, lateral wall and modiolus of chinchilla showed enrichment of cell death pathways in both the sensory epithelia and modiolus [54]. Immunolabeling experiments confirmed the antibody microarray results by identifying the expression of E2F3 in nuclei and WSTF and FAK-p-Tyr577 in the stereocilia in the organ of Corti. Detection of FAK-p-Tyr577 supports the idea that post-translational modification of proteins can be identified using antibody microarrays, but cannot be detected using gene arrays [54]. It is necessary to have a large amount of tissue to study proteomics, and this problem was circumvented by an approach called “Subtractive Strategy Using Mouse Mutants” that can detect large number of proteins with only a small amount of starting material. Using the *Pou4f3* mutant mouse model, the association of *Pou4f3* in the inner ear with other genes, such as *Notch1*-*4, gata3*, *p27Kip1*, *Eya1*, *S100a1*, *Tbx3*, *Shh*, *Fgfr3*, *Bmp5*, *Jag2* and *Fkh10*, were able to be identified [50]*.*

### 5.2. Liquid Chromatography Coupled with Mass Spectrometry

Recently, a study by Peng* et al.* [55] demonstrated the ability of liquid chromatography coupled with tandem mass spectrometry (LC-MS/MS) to detect hundreds of proteins in the normal cochlea of mice. LC-MS/MS can be used to detect protein expression and PTMs, and this study detected 628 proteins, the largest number of proteins identified in the organ of Corti, so far. Using the same LC-MS/MS technique, another study identified 620 and 134 proteins in the embryonic stage (E20–E21) chicken utricle and cochlea, respectively [57]. Organ of Corti from the normal mice showed expression of many proteins, such as cochlin, isoform-1-α-tectorin, gap junction β6 protein and myosin VI, which are all involved in hearing impairment. Both phosphorylated and acetylated forms of proteins, including both mono- and di-phosphorylated states and N-termini and lysine acetylated states, could be detected using LC-MS/MS [55]. Calcium buffers were abundant in the cochlea, while histones and nuclear lamins were abundant in the utricle. Heat shock proteins were found to be of equal abundance in both cochlea and utricle of chicken. Validation of mass spectrometry (MS) data was done on a subset of proteins using immunoblots, and it showed similar trends in the differential abundance between utricle and cochlea, as was shown by MS. Differential expression of glycolytic and gluconeogenesis enzymes were high in cochlea, while citric acid cycle and electron transport chain enzymes were high in utricle. In addition to this, glucose and lactate transporters were upregulated in cochlea identified by MS. Immunoblot and immunocytochemistry validated the MS data, and moreover, glycolytic rates measured using tritiated hydrogen also confirmed the high energy demand in the cochlea [57]. 

Another study using LC-MS/MS identified many presynaptic proteins in the ribbon synapses [56] that are present in hair cells that exhibit fast kinetics and are optimized to release large amounts of neurotransmitters [94]. The presynaptic proteins can be categorized into vesicle and membrane transport proteins, proteins that regulate synaptic exocytosis, ion channels, transporters, pumps and calcium binding proteins. Immunoblots and immunofluorescence labeling confirmed the expression of SNAP25, NSF, syntaxin1, syntaxin6, VAMP2, alpha α-SNAP, β-SNAP and VAP33 proteins in the chicken cochlear hair cells. The proteomic profile of the synaptic fraction showed that otoferlin, synaptotagmin7, alpha-synuclein, syntaphilin, piccolo, synaptojanin2 and SCAMP1 were only identified in the cochlea of chicken, but not in the retina or brain, suggesting these proteins have specific function in hair cells and that there are compositional differences between hair cell and retinal ribbon synapses [94]. The proteome of hair cell bundles in chicken utricles detected 59 proteins, representing a large fraction of cytoskeletal, energy metabolism and stress response proteins and other proteins, such as calcium buffers and transmembrane proteins. Both actin and creatine kinase B were found to be abundant, and using quantitative immunoblot, actin was shown to be eight-fold higher than creatine kinase B (B-CK). Immunolabeling confirmed B-CK expression in chicken and bullfrog utricles and in mouse inner and outer hair cells [56]. 

In conclusion, these studies show the potential of proteomics to identify proteins that cannot be identified from mRNA expression levels alone. Antibody microarrays can be customized to include antibodies to detect target proteins of interest that can have specific PTMs. The use of proteomics to investigate biological questions in the inner ear has been limited, due to the requirement of a large amount of tissue, and inner ears are very small and often encased in bony structures. This problem was solved by the development of the subtractive strategy [49] and LC-MS/MS methods [55,56,57], which can use minute quantities of starting materials and detect hundreds of proteins.

## 6. Conclusions and Perspectives

The development of technologies that allow high throughput gene expression analysis at a relatively low cost has led to an increase of microarray and, now, RNA-seq studies related to the inner ear. These studies have resulted in massive amounts of gene expression data. In the publications resulting from these studies, authors only have space to focus on a few of the significantly regulated genes and/or cellular pathways. Fortunately, a requirement for publication of gene expression data in most journals today is the submission of all the data in a public database, such as the Gene Expression Omnibus (GEO) at the National Center for Biotechnology Information (NCBI). Similar repositories are available for the submission of proteomics data. Unfortunately, because of differences in species, tissues, treatments and developmental stages (information that is submitted along with the gene expression data), it is not easy to compare gene expression patterns across multiple studies. Bioinformaticians are needed to help develop standards for performing meta-analyses with multiple gene expression datasets, so that the most conserved and important pathways related to hair cell regeneration can be focused upon.

In addition, it is clear that transcriptomics does not give a complete picture of the workings of the proteins in a cell, as it misses post-transcriptional and post-translational modifications. Future progress in understanding the processes of hair cell regeneration will rely on collaborations and integration between genomics, transcriptomics, miRNA analysis, proteomics and bioinformatics.

Hopefully, such collaborations will lead to the development of therapeutics that can prevent hair cell loss or promote hair cell regeneration in humans. Indeed, there is evidence that genes found to be regulated in the inner ear via microarray studies can lead to translational research. One example is a recent clinical trial using insulin growth factor-1 (IGF-1) following hearing loss. Growth hormone (GH) has been shown to be regulated in the inner ear of mice during development [42] and in zebrafish following trauma [25]. One of the main targets of GH is IGF-1, an insulin-like growth factor; binding proteins, *IGFBP-2* and *IBFBP-6,* are expressed in the rat cochlea at greater levels than the central nervous system [58], and *IGFBP-2* and *IBFBP-5* are regulated during cochlear cell differentiation [40]. In addition, IGF-1 null mice have developmental abnormalities in the inner ear, suggesting that it plays a vital role in development [66]. Trials with noise-exposed guinea pigs showed that application of recombinant IGF-1 via a gelatin hydrogel applied to the round window membrane of the ear resulted in increased survival of outer hair cells and reduced hearing thresholds [95]. Use of gelatin hydrogels was a novel vehicle for delivering growth factors to the inner ear for clinical application. More recently, this same protocol has been applied in human clinical trials. Patients with sudden sensorineural hearing loss had gelatin hydrogels impregnated with IGF-1 applied to the middle ear. Forty-eight percent and 56% of the patients showed hearing improvement after 12 and 24 weeks, respectively [96]. Follow-up research examining mechanisms of the effects of this growth factor found that IGF-1 inhibits hair cell apoptosis and promotes cell proliferation in supporting cells in the mouse ear [97]. Although there was considerable variability in the results of the IGF-1 clinical trial, it demonstrates how high throughput molecular analyses can lead to testing in mammalian models followed by human clinical trials. 
